# Pharmacophore Identification and QSAR Studies on Substituted Benzoxazinone as Antiplatelet Agents: kNN-MFA Approach

**DOI:** 10.3797/scipharm.1112-09

**Published:** 2012-02-26

**Authors:** Prafulla B. Choudhari, Manish S. Bhatia, Swapnil D. Jadhav

**Affiliations:** Drug Development Sciences Group, Department of Pharmaceutical Chemistry, Bharati Vidyapeeth College of Pharmacy, Kolhapur Maharashtra, 416013, India

**Keywords:** QSAR, Anti-platelet, Drug design, kNNMFA

## Abstract

The three-dimensional quantitative structure–activity relationship (3D-QSAR) and pharmacophore identification studies on 28 substituted benzoxazinone derivatives as antiplatelet agents have been carried out. Multiple linear regression (MLR) method was applied for QSAR model development considering training and test set approaches with various feature selection methods. Stepwise (SW), simulated annealing (SA) and genetic algorithm (GA) were applied to derive QSAR models which were further validated for statistical significance and predictive ability by internal and external validation. The results of pharmacophore identification studies showed that hydrogen bond accepters, aromatic and hydrophobic, are the important features for antiplatelet activity. The selected best 3D kNN-MFA model A has a training set of 23 molecules and test set of 5 molecules with validation (q^2^) and cross validation (pred_r^2^) values 0.9739 and 0.8217, respectively. Additionally, the selected best 3D QSAR (MLR) model B has a training set of 23 molecules and test set of 5 molecules with validation (r^2^) and cross validation (pred_r^2^) values of 0.9435 and 0.7663, respectively, and four descriptors at the grid points S_123, E_407, E_311 and H_605. The information rendered by 3D-QSAR models may lead to a better understanding and designing of novel potent antiplatelet molecules.

## Introduction

Cardiovascular and other vascular diseases like cerebrovascular diseases attract much attention in the realm of medical and drug research due to their threat as a main cause of morbidity and mortality. The platelet aggregation is an important process in healing and is also an important pathogenetic factor in the CVS diseases. The rapid occlusion of an arterial vessel by formation of a thrombotic plug is the crucial event leading to hypoxia in the brain. Platelets play a major role in hemostasis but also in arterial thrombosis. Because of the limited effectivity of currently used antiplatelet drugs like aspirin and ticlopidine, serious thromboembolic complications are occurring, so the designing of new and novel antiplatelet agents is becoming the area of choice for various researchers. QSAR approach [1–10] is certainly useful for drug design for both known and unknown targets. The molecular descriptors are calculated from the chemical structures of the molecules so that these can be utilised for deriving the relationships between the activity and molecular properties. QSAR substantially increases the potential of work, avoiding time and resource consuming experiments. The improvement in three-dimensional structural information (3D) of bioorganic molecules with fast alignment has led to the development of 3D descriptors which are associated with 3D-QSAR methods. Moreover, QSAR approaches that employ 3D descriptors have been developed to address the problems of 2D-QSAR techniques, such as their inability to distinguish stereoisomers. The present article is an attempt to develop QSAR models based on three-dimensional quantitative structure–activity relationship (3D-QSAR) methods for benzoxazinone compounds.

## Results and Discussion

In the present study 3D QSAR models by kNN-MFA [2–4] are developed coupled with stepwise variable selection method, and Multiple linear regression (MLR) are developed for benzoxazinone derivatives based on steric, electrostatic and hydrophobic fields. The descriptors that get selected in a given model are the field points either of steric, electrostatic and hydrophobic nature at particular locations in a common grid around a reported set of molecules. The field values of compounds in the cluster of most active compounds decide the range of field values which is preferred and recommended for new compound design.

### Interpretation of 3QSAR Model (MLR) [5–10]

**Fig. 1.**
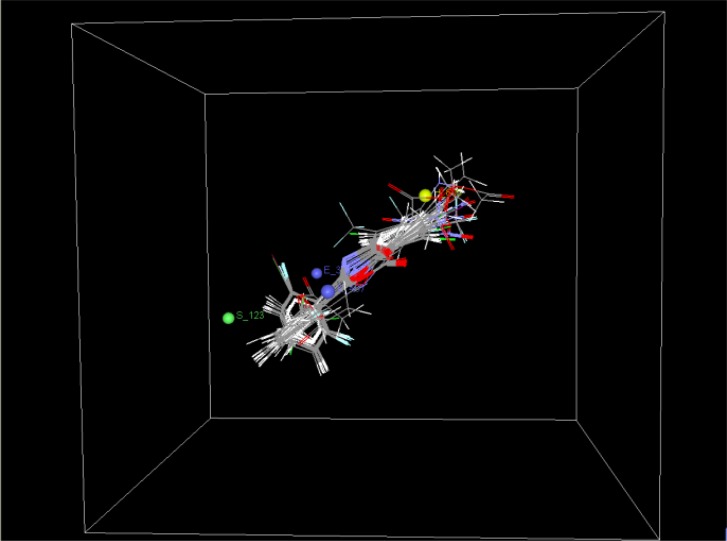
Field point for selected QSAR model A

**Fig. 2.**
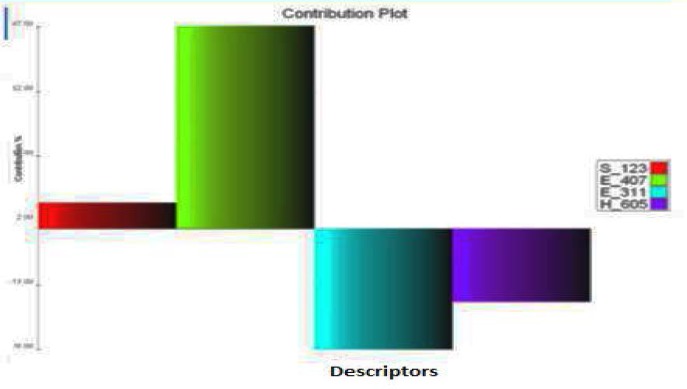
Contribution plot for selected QSAR model A

**Fig. 3.**
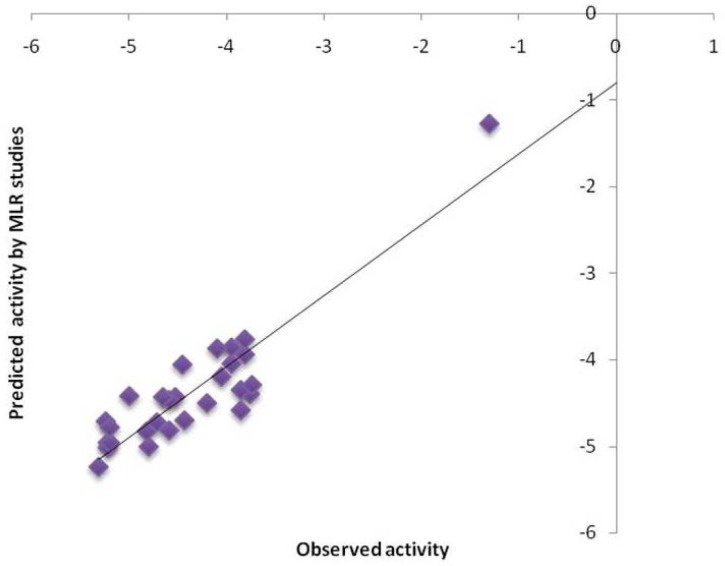
Correlation plot for selected QSAR model A

The structural requirement of the benzoxazinone analogs to show anti-platelet activity is elaborated by the MLR studies. The two different 3D QSAR models from the MLR studies that are obtained are model A and B. The model A is selected on the basis of statistical significance. The model A has correlation coefficient (r^2^) 0.9435 (Table 1), as compared to that of model B (0.8780). In model A S_123, E_407, E_311, H_605 (Figures 1, 2 and 3) which are the steric, electrostatic and hydrophilic field energies of interactions between probe (CH_3_) with charge +1 and compounds at their corresponding spatial grid points of 123, 407, 311 and 605. The steric and electrostatic grind point at 407 and steric grid point at 123 have positive contributions of 47% and 2%, respectively, while electrostatic and hydrophilic grind point at 311 and 605 have negative contributions of 30% and 21%, respectively. The electrostatic interaction at lattice point E_311, H_605 are negatively contributing, which means substitution of electron withdrawing groups on the aryl ring of benzoxazinone can increase the antiplatelet activity. Furthermore, the hydrophobic interaction at the lattice point 605 is also negatively contributing, which means the substitution at the R1 should be less hydrophobic, and the decrease in chain length could increase the activity. The Electrostatic interaction at the lattice point 407 and steric interaction at lattice point 123 are positively contributing so the mono substitution of on electron releasing groups at the ortho position (R2) can increase the activity (Table 2). Also, the substitution of more bulky groups or larger groups such as methoxy and benzoyl can increase the activity by keeping the benzoxazinone ring in perpendicular plane to the other aryl ring.

### Interpretation of 3QSAR Model (kNN-MFA)

**Fig. 4.**
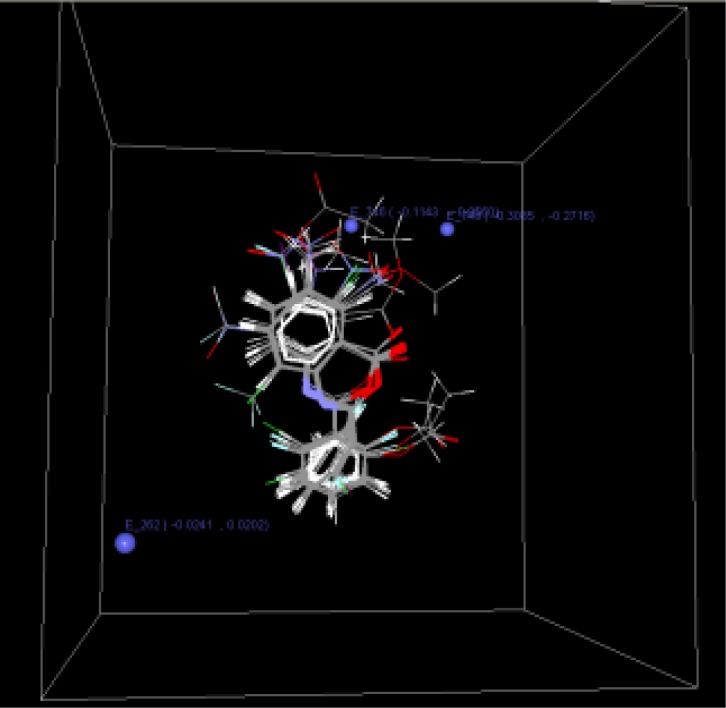
Field point for selected QSAR model C

Model C is the second model which is selected on the basis of statistical coefficient like q^2^ (0.9739) and Pred r^2^ (0.8217)(Table 3). The contributing descriptors for this model are E_746 (−0.1143...−0.0560), E_748 (−0.3085...−0.2716) (Figure 4) which indicates that substitution involving electron deficient group is preferred for substitution at R1, the nitro substituted compound can show potent activity. That the range at the lattice point E_262 (−0.0241...0.0202) is positive indicates substitution with more electron density could yield more active molecules (Table 4). The results of kNN-MFA methods show similar results to the MLR studies which indicates that these two methods can be utilized to validate each other (Figure no 5).

### Pharmacophore identification studies using Vlife MDS 3.5 [10]

**Fig. 6.**
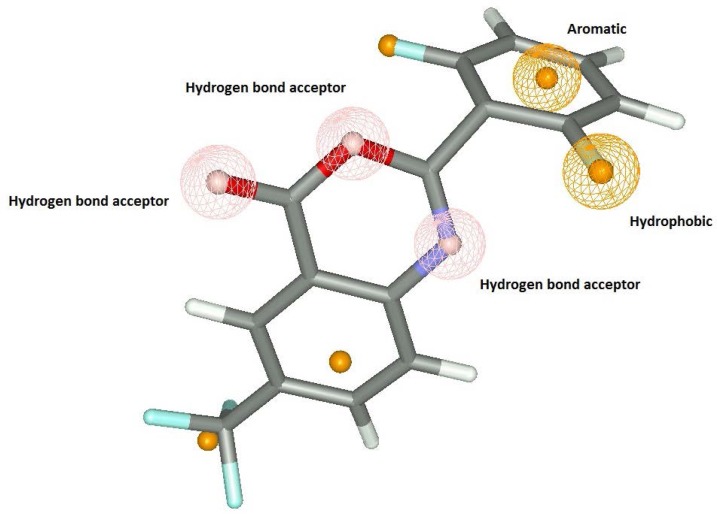
Selected pharmacophore model

The pharmacophore identification studies are carried out in Mol sign module of Vlife MDS 3.5. Pharmacophore is a three-dimensional description of the features needed for activity. These features include hydrogen bond donors and acceptors, aromatic groups, bulky hydrophobic groups, positively ionisable and negatively ionisable. The pharmacophoric features important for antiplatelet activity are hydrogen bond acceptors, hydrophobic groups and hydrophilic groups (Figure 6). The three hydrogen bond acceptors must be at least 2.27 Å and 3.984 Å apart from each other. The hydrophobic and hydrogen bond acceptors are 4.050 Å. The compounds to show the anti-platelet activity must have these features in their structures.

## Conclusion

In this work we indentified structural requirements of benzoxazinones to act as antiplatelet agents. The QSAR models generated by MLR and kNN-MFA show similar results. Thus, kNN-MFA technique can be utilized as a tool for drug design.

## Experimental

### Computational details

#### Dataset

A dataset of 28 compounds was taken from the published antiplatelet derivatives by Katritzky et.al [11]. The structures and their inhibitory activities in logIC50 are listed in Table 5.

## Materials and methods

### Ligand Preparation

The structure of benzoxazinone was used as the template to build the molecules in the dataset in Vlife MDS 3.5. The structure was minimized using the standard Merck molecular force field (MMFF) with distance dependant dielectric function and energy gradient of 0.001 kcal/mol Å.

### Molecular alignment

**Fig. 7.**
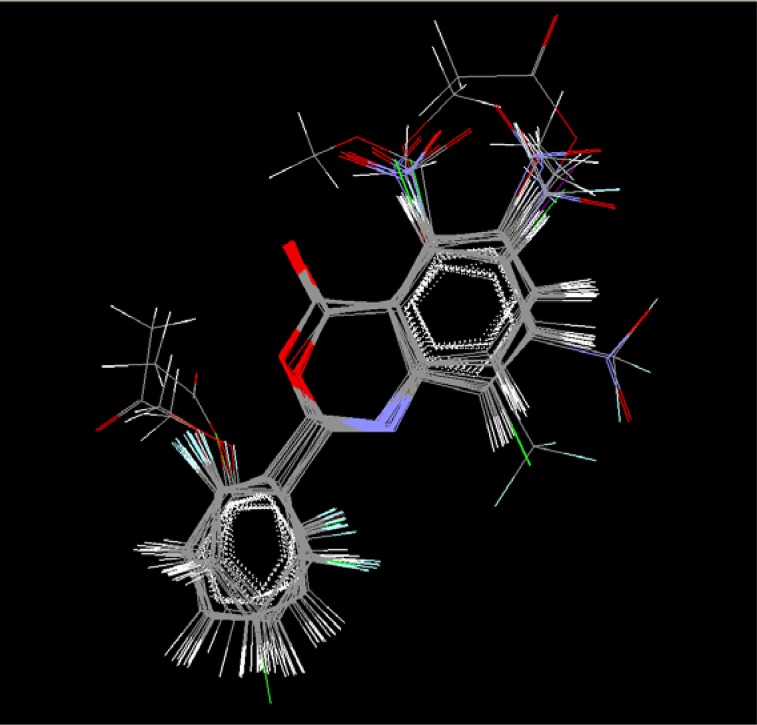
Alignment of the molecules

The molecules of the dataset were aligned by the template based technique, using the common structure of benzoxazinone. The most active molecule was selected as a template for alignment of the molecules. The alignment of all the molecules on the template is shown in (Figure 7)

### Descriptor Calculation

Like many 3D QSAR methods, a suitable alignment of a given set of molecules was performed using the Vlife MDS 3.5 Engine. This was followed by generation of a common rectangular grid around the molecules. The hydrophilic, steric and electrostatic interaction energies are computed at the lattice points of the grid using a methyl probe of charge +1. These interaction energy values are considered for relationship generation and utilized as descriptors to decide nearness between molecules. The term descriptor is utilized in the following discussion to indicate field values at the lattice points. The molecules under study were divided into test set and training set randomly.

### 3D QSAR studies using multiple linear regression

#### Stepwise multiple regression (SMR)

It is an approach to select a subset of variables when the numbers of independent variables (descriptors) are much more than the number of data points (molecules). SMR is a way of computing OLS regression in stages. It is also a procedure to examine the impact of each variable to the model step by step. Each variable is added to the equation and a new regression is performed. The variable that cannot contribute much to the variance explained would not be added. As a result, SMR generates a single multiple regression equation.

### 3D QSAR Studies using kNN MFA

The calculated fields of the randomly selected 23 molecules used in the training set were considered as observations to generate QSAR equations using a stepwise variable selection (SW) kNN MFA method. Plot of the kNN MFA which shows the relative position and ranges of the corresponding important electrostatic/steric fields in the model provides the following guidelines for design of new molecules.

### Pharmacophore modeling

Pharmacophore modeling was carried out using the mol sign module of Vlife MDS 3.5 software. Series of platelet inhibitors were first aligned on the active molecule. A pharmacophore model is a set of three-dimensional features that are necessary for bioactive ligands. Thus, it makes logical sense to align molecules based on features that are responsible for bioactivity. The software was set to generate a minimum of 4 pharmacophoric features keeping the tolerance distance at 10 Å.

## Figures and Tables

**Fig. 5.**
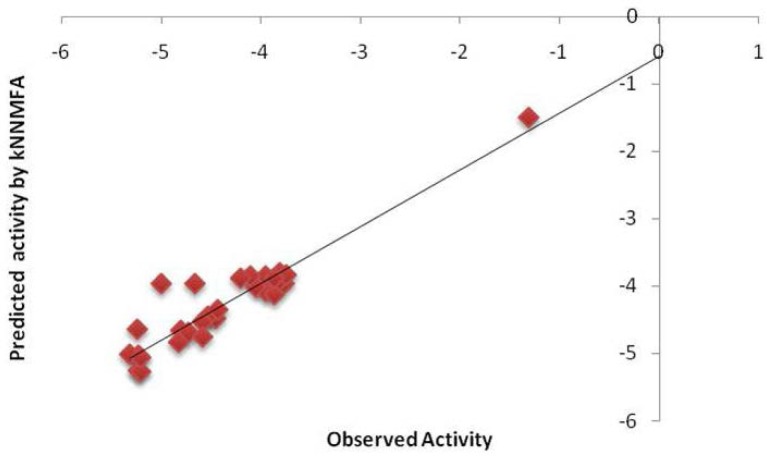
Correlation plot for selected QSAR model C

**Tab. 1.** Selected MLR QSAR equations along with statistical parameters employed for model selection.

**Model No.**	**QSAR model**	**N**	**r2**	**q2**	**F value**	**Pred r2**
A	pIC50=0.0036+11.7432(±5.4497) S_123+11.7432(±5.4497) E_407-1.3306(±0.2655) E_311-2.0181(±0.6882) H_605	28	0.9435	0.8784	64.2607	0.7663
B	pIC50=0.0014+1.9224(±0.6960) E_735-4.3727(±0.1702) H_305+0.8246(±0.1221) E_708+1.0651(±0.2229) S_794	28	0.8780	0.7365	32.3774	0.7489

**Tab. 2.** Observed and predicted activity for Model A

**Sr no**	**Observed activity**	**Predicted activity**	**Residuals**
1	−4.796	−5.009	0.213
2	−3.951	−4.048	0.097
3	−5.222	−4.957	−0.264
4	−4.721	−4.729	0.008
5	−3.76	−4.395	0.635
6	−3.813	−3.939	0.126
7	−4.456	−4.052	−0.403
8	−3.813	−3.761	−0.051
9	−3.951	−3.859	−0.091
10	−4.051	−4.193	0.142
11	−3.86	−4.348	0.488
12	−4.097	−3.875	−0.221
13	−5	−4.422	−0.577
14	−4.824	−4.827	0.003
15	−4.201	−4.496	0.295
16	−5.237	−4.707	−0.529
17	−4.523	−4.430	−0.092
18	−4.585	−4.822	0.237
19	−1.31	−1.275	−0.034
20	−4.432	−4.701	0.269
21	−5.222	−5.018	−0.203
22	−3.745	−4.297	0.552
23	−3.86	−4.581	0.721
24*	−4.585	−4.471	−0.113
25*	−5.31	−5.240	−0.069
26*	−5.201	−4.957	−0.243
27*	−5.201	−4.778	−0.422
28*	−4.658	−4.435	−0.222

* ...Test set molecules.

**Tab. 3.** Selected kNNMFA QSAR equations along with statistical parameters employed for model selection.

**Model No.**	**Selected Descriptors**	**N**	**Descriptor Range**	**q2**	**Pred r2**	**Degree of freedom**
	E_746		E_746 (−0.1143...−0.0560)			
C	E_262	28	E_262 (−0.0241...0.0202)	0.9739	0.8217	19
	E_748		E_748 (−0.3085...−0.2716)			
D	E_295	28	E_295 (2.7514...5.7547)	0.7425	0.6427	20
	E_235		E_235 (−0.6487...0.1711)			

**Tab. 4.** Observed and predicted activity for Model C

**Sr no**	**Observed activity**	**Predicted activity**	**Residuals**
1	−4.796	−4.654	−0.141
2	−3.951	−3.853	−0.097
3	−5.222	−5.253	0.031
4	−4.721	−4.691	−0.029
5	−3.76	−3.954	0.194
6	−3.813	−3.909	0.096
7	−4.456	−4.476	0.020
8	−3.813	−3.802	−0.010
9	−3.951	−4.076	0.125
10	−4.051	−4.008	−0.042
11	−3.86	−4.123	0.263
12	−4.097	−3.856	−0.240
13	−5	−3.955	−1.044
14	−4.824	−4.832	0.008
15	−4.201	−3.885	−0.315
16	−5.237	−4.633	−0.603
17	−4.523	−4.444	−0.078
18	−4.585	−4.521	−0.063
19	−1.31	−1.491	0.181
20	−4.432	−4.345	−0.086
21	−5.222	−5.029	−0.192
22	−3.745	−3.835	0.090
23	−3.86	−3.884	0.024
24*	−4.585	−4.758	0.173
25*	−5.31	−5.010	−0.299
26*	−5.201	−5.262	0.061
27*	−5.201	−5.054	−0.146
28*	−4.658	−3.955	−0.702

* ...Test set molecules.

**Tab. 5.** Structure of studied molecules

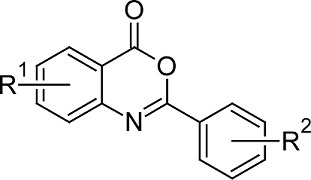			

**Sr No**	**R^1^**	**R^2^**	**Observed activity (logIC50)**
1	6-CF_3_	2,6-F	−4.796
2	7-NO_2_	2,6-F	−3.951
3	5-F	2,6-F	−5.222
4	6-NO_2_	2,6-F	−4.721
5	7-CF_3_	2,6-F	−3.76
6	6-OCH_3_	2,6-F	−3.813
7	6-NHAc	2,6-F	−4.456
8	6-NH_2_	2,6-F	−3.813
9	5-COOCH_3_	2,6-F	−3.951
10	5-CH_3_	2,6-F	−4.051
11	H	2-F	−3.86
12	8-CF_3_	2,6-F	−4.097
13	6-CH_3_	2,6-F	−5
14	6-I	2-Cl	−4.824
15	6-CH_3_	2,6-Cl	−4.201
16	5-NO_2_	2-OCH_3_	−5.237
17	H	2-OCH_3_,5-Cl	−4.523
18	5-NO_2_	2-COOMe	−4.585
19	6-NO_2_	2-COOMe	−1.31
20	6-CF_3_	2-F	−4.432
21	6-Cl	2-Br	−5.222
22	5,8-Cl	2-F	−3.745
23	5-COOCH_3_	2-F	−3.86
24	5-NO_2_	2-F	−4.585
25	5-Cl	2,6-F	−5.31
26	5-NO_2_	2,6-F	−5.201
27	5,8-Cl	2,6-F	−5.201
28	6-CH_3_	2,6-F	−4.658
